# A cellular model for the investigation of depot specific human adipocyte biology

**DOI:** 10.1080/21623945.2016.1277052

**Published:** 2017-01-06

**Authors:** Marijana Todorčević, Catriona Hilton, Catriona McNeil, Constantinos Christodoulides, Leanne Hodson, Fredrik Karpe, Katherine E. Pinnick

**Affiliations:** a Oxford Centre for Diabetes, Endocrinology and Metabolism, Radcliffe Department of Medicine, University of Oxford, Oxford, UK; b NIHR Oxford Biomedical Research Centre, OUH Trust, Churchill Hospital, Oxford, UK

**Keywords:** abdominal, adipogenesis, body fat distribution, gluteal, human adipose tissue, preadipocyte cell lines

## Abstract

Upper-body adiposity is associated with increased metabolic disease risk, while lower-body adiposity is paradoxically protective. Efforts to understand the underlying mechanisms require appropriate and reproducible *in vitro* culture models. We have therefore generated immortalised (_im_) human preadipocyte (PAD) cell lines derived from paired subcutaneous abdominal and gluteal adipose tissue. These cell lines, denoted _im_APAD and _im_GPAD display enhanced proliferation and robust adipogenic capacities. Differentiated _im_APAD and _im_GPAD adipocytes synthesize triglycerides *de novo* and respond lipolytically to catecholamine-stimulation. Importantly the cells retain their depot-of-origin ‘memory’ as reflected by inherent differences in fatty acid metabolism and expression of depot-specific developmental genes. These features make these cell lines an invaluable tool for the *in vitro* investigation of depot-specific human adipocyte biology.

## Introduction

Obesity is an escalating public health problem which underpins several metabolic complications including type 2 diabetes and cardiovascular disease.^1,2^ Different regional fat depots have distinct functional traits and diverse associations with metabolic disorders. Increased waist-to-hip ratio (WHR) is an indicator of preferential upper-body fat accumulation (abdominal subcutaneous and/or visceral). A high WHR is generally more closely associated with obesity-related metabolic disease risk than measures of overall obesity, i.e. body mass index (BMI).^3-5^ By comparison, fat accumulation in the lower-body (gluteal, femoral) is paradoxically associated with reduced cardiometabolic risk after adjustment for BMI and considered beneficial.^3,6,7^ Human body fat distribution is a heritable trait which displays a strong degree of sexual dimorphism.^8-10^ Recent GWAS meta-analyses have identified multiple genetic loci associated with measures of body fat distribution (waist, hip and WHR), independent of BMI.^11,12^ Many of these loci are located within and/or near genes implicated in adipocyte development or function. Despite this the molecular mechanisms that determine human body fat patterning, and which link adipocyte biology, body fat distribution and metabolic disease risk, remain poorly understood. This has led to a growing need to establish suitable *in vitro* models to facilitate the investigation of regional adipocyte biology.

White adipose tissue (WAT) has traditionally been viewed as a site of energy storage and release, though WAT is now also increasingly recognized as a complex endocrine organ.^13^ However, not all WAT is alike.^14,15^ WAT depots from different regional sites in the human body exhibit distinct functional properties relating to: lipid storage^16,17^ and turnover,^18,19^ adipokine secretion,^20,21^ and inflammation.^22,23^ Transcriptional profiling of WAT, to identify depot-specific gene expression, has demonstrated a strong enrichment for developmental genes involved in embryological patterning,^24-27^ suggesting different WAT depots have divergent developmental origins.^28^ Similar depot-specific transcriptional profiles are also observed in isolated adipocyte precursors (preadipocytes).^29^ These depot-specific expression profiles are intrinsic and are retained across multiple preadipocyte generations when sub-cultured *in vitro*.^27,29^ Furthermore, preadipocytes differentiated *in vitro* retain many of the functional traits of their depot of origin e.g. lipolytic activity, fatty acid metabolism, and adipokine secretion.^30-32^ In addition they exhibit different cellular dynamics including rates of replication, adipogenic capacity, and sensitivity to apoptotic stimuli.^33,34^

A prerequisite for an *in vitro* model to aid the study of body fat distribution is the ability to examine preadipocytes from more than one WAT depot in parallel. This requirement is not met by any of the currently available rodent or human preadipocyte cell lines (e.g., 3T3-L1, Simpson-Golabi-Behmel-Syndrome (SGBS) or ChubS7 cell lines).^35-37^ In this study we report the successful generation of immortalised (_im_) human preadipocyte (PAD) cell lines derived from paired abdominal subcutaneous (ASAT) and gluteal subcutaneous adipose tissue (GSAT), referred to herein as _im_APAD and _im_GPAD, respectively. The _im_APAD and _im_GPAD cell lines display enhanced proliferation rates compared with primary cells isolated from the same donor (1°APAD and 1°GPAD). Furthermore, they retain the capacity for terminal adipogenic differentiation, *de novo* lipogenesis (DNL) and catecholamine-stimulated lipolysis. Finally, they possess inherent gene expression signatures that mirror those of 1°APAD and 1°GPAD human preadipocytes. To our knowledge this represents the first example of paired human preadipocyte cell lines derived from abdominal and gluteal subcutaneous adipose tissue.

## Results

### Generation of hTERT and HPV16-E7 co-expressing human preadipocyte cell lines

**Figure 1.**
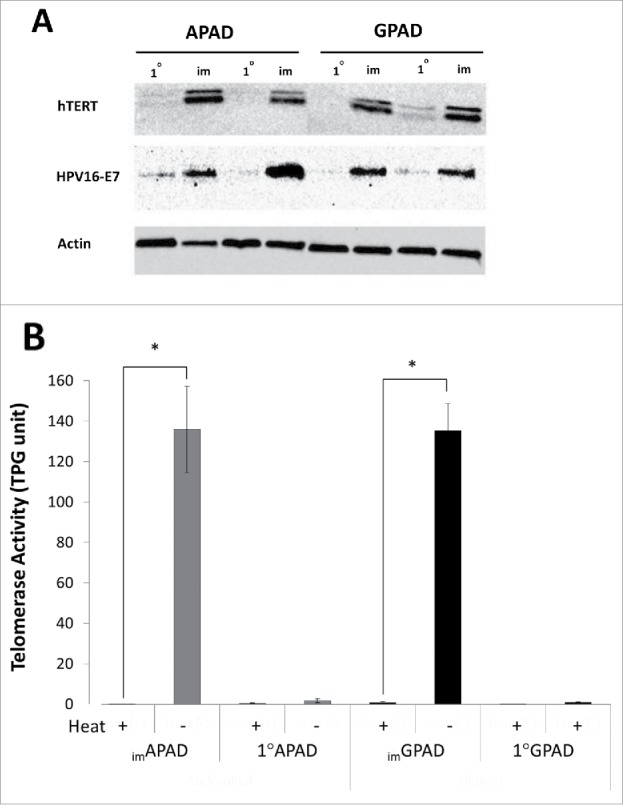
Overexpression of hTERT and HPV16-E7 in _im_APAD and _im_GPAD cell lines. (A) overexpression of hTERT and HPV16-E7 protein was confirmed by Western blotting in the paired _im_APAD and _im_GPAD cell lines (passage 15–17) and compared with 1°APAD and 1°GPAD preadipocytes (passage 6) from the same donor. Labeling for actin is shown as a loading control. (B) Telomerase activity was determined in _im_APAD and _im_GPAD cell lines (passage 11) and 1°APAD and 1°GPAD cells (passage 6) (n = 3, mean ± SEM; *P < 0.05, paired samples *t*-test). Heat treatment was used to inactivate telomerase as a negative control.

To generate the _im_APAD and _im_GPAD cell lines paired 1°APAD and 1°GPAD cells, originating from the same male donor, were transduced with lentiviral particles carrying the human telomerase (hTERT) gene and the human papillomavirus type-16 E7 oncoprotein (HPV16-E7). Protein expression of hTERT and HPV16-E7 was confirmed in the _im_APAD and _im_GPAD cell lines by Western blot analysis (Fig. 1A). hTERT and HPV16-E7 protein activity was over 100-fold higher in _im_APAD and _im_GPAD cell lines than that observed in the 1°APAD and 1°GPAD cells (Fig. 1B). Collectively these data confirmed the successful overexpression of hTERT and HPV16-E7 in the _im_APAD and _im_GPAD cell lines.

### Proliferative capacity of _im_APAD and _im_GPAD cell lines

**Figure 2.**
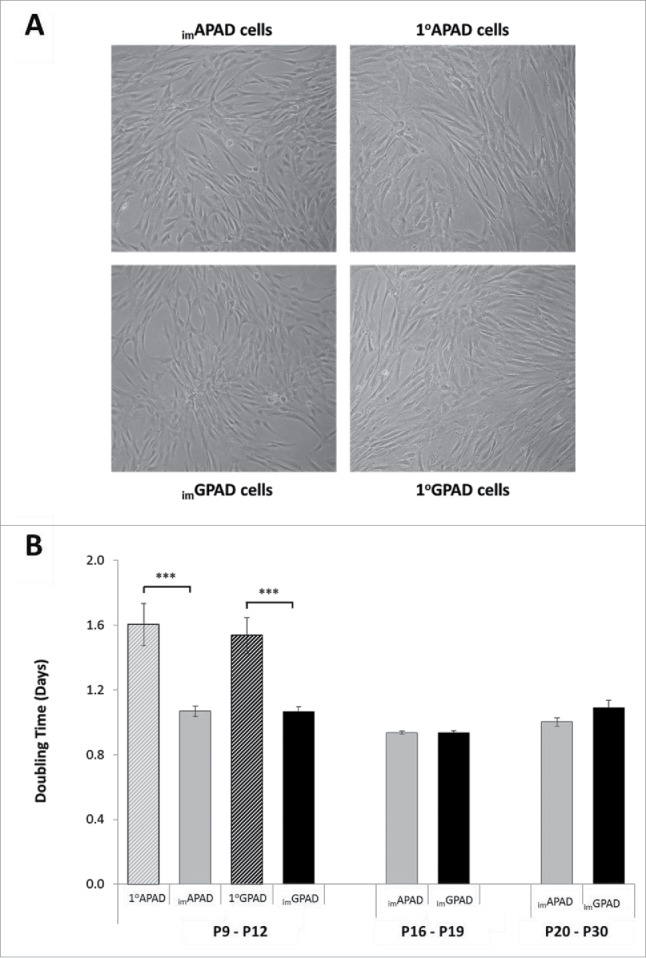
Proliferation of _im_APAD and _im_GPAD cell lines. (A) Light microscopy of proliferating _im_APAD and _im_GPAD cell lines compared with 1°APAD and 1°GPAD cells (x 100 magnification). (B) Cell doubling time of paired _im_APAD/_im_GPAD cell lines was compared with 1°APAD/1°GPAD cells (passage 9–12). Proliferation rates were examined up to passage 30 for _im_APAD/_im_GPAD cells but 1°APAD and 1°GPAD cells failed to proliferate after passage 14 (n = 6–8, mean ± SEM; ***P < 0.001; paired samples *t*-test for cell line vs. primary cells).

Proliferating _im_APAD and _im_GPAD cell lines displayed a fibroblast-like morphology and were visually comparable to 1°APAD and 1°GPAD cells (Fig. 2A). Proliferation rates of the immortalised cell lines were compared with those of the primary cells (Fig. 2B). Both sets of cells were plated at a low density and cultured for 4 d before counting. Significantly higher cell counts were obtained for the _im_APAD and _im_GPAD cell lines; the mean doubling time of _im_APAD and _im_GPAD cell lines (passages 9–12) was 1.1 ± 0.03 d for both cell lines, compared with 1.6 ± 0.13 d and 1.5 ± 0.11 d for the 1°APAD and 1°GPAD cells, respectively. The proliferation rates of _im_APAD and _im_GPAD cells were equivalent and no significant depot difference was observed between the 2 cell lines (*P* = 0.18). At passage 14 the 1°APAD and 1°GPAD cells became senescent and failed to proliferate despite extending the culture period to 7 d (Supplementary Fig. 1) and further comparisons between the immortalised cell lines and primary cells were not possible. In contrast, the _im_APAD and _im_GPAD cell lines retained their proliferative capacity up to passage 30 with mean doubling times of 1.0 ± 0.03 and 1.1 ± 0.05, respectively (Fig. 2B).

### Transcriptional profiles of _im_APAD and _im_GPAD cell lines during adipogenic differentiation

**Figure 3.**
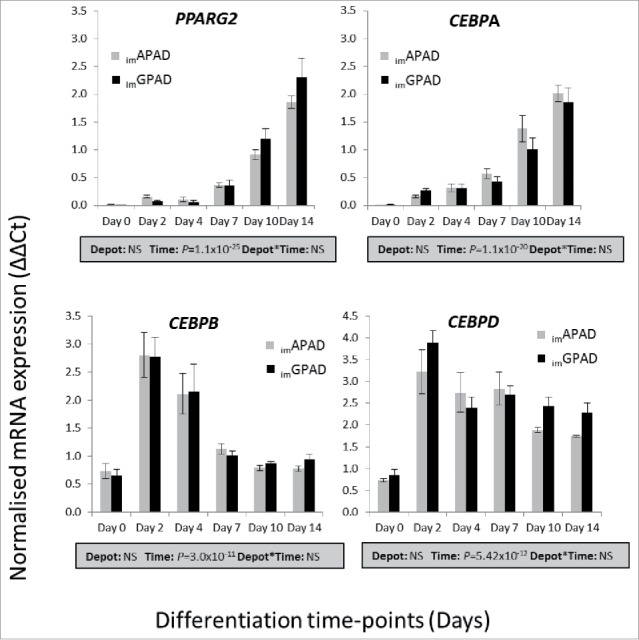
mRNA expression of adipogenic transcription factors in _im_APAD and _im_GPAD cell lines during adipogenesis. mRNA expression of *PPARG2, CEBPA, CEBPB* and *CEBPD* over a 14 day adipogenic differentiation time-course was determined in _im_APAD and _im_GPAD cells (passage 8–9) by real-time qPCR. Data are shown as ΔΔCt values (normalized to *PPIA* and *PGK1*; n = 6, mean ± SEM). A multivariate general linear model was used to test for statistical significance between depots and time, and to assess depot × time interactions. *P*-values are presented in the shaded boxes, NS: non-significant.

The phenotypic and functional changes that occur during the transition of preadipocytes to differentiated adipocytes are under the regulation of several key transcription factors which include CEBPA, CEBPB, CEBPD and PPARG2. The mRNA expression of these transcriptional regulators were assessed over a 14-day adipogenic time-course in the _im_APAD and _im_GPAD cell lines (passages 8–9) and compared with 1°APAD and 1°GPAD cells (passages 6–8). The expression profiles of _im_APAD and _im_GPAD cells (Fig. 3) mirrored those of the 1°APAD and 1°GPAD (Supplementary Fig. 2) for all selected genes. As expected, expression of *CEBPA* and *PPARG2* increased steadily throughout the differentiation period in all cells with maximal expression observed between days 10 and 14. By comparison, *CEBPB* and *CEBPD* exhibited a more rapid induction, peaking at day 2, and subsequently declining over the remainder of the time-course. Expression of the adipogenic transcription factors was equivalent between _im_APAD and _im_GPAD cells. Overall, these data indicate that, at the mRNA level, the _im_APAD and _im_GPAD cell lines retain the basic machinery required for successful white adipogenic differentiation. Furthermore, *UCP1*, a marker for brown and beige adipocytes showed essentially no expression at day 14 in both _im_APAD (ΔΔCt: 0.27 ± 0.07) and _im_GPAD (ΔΔCt: 0.24 ± 0.04) cells compared with the high level of *UCP1* expression observed in differentiated primary human brown adipocytes at day 14 (ΔΔCt: 44.9 ± 11.7). To confirm that white adipogenic capacity was retained in higher passage generations of the _im_APAD and _im_GPAD cell lines, gene expression analyses were performed in passage 17–21 cells (Supplementary Fig. 3). As seen in early passage cells, both cell lines displayed strong induction of *PPARG2* and *CEBPA* over the differentiation time-course with maximal levels observed at day 14. Expression levels were equivalent with no differences in expression between the _im_APAD and _im_GPAD cell lines.

### Intracellular lipid accumulation in _im_APAD and _im_GPAD cell lines following adipogenesis

**Figure 4.**
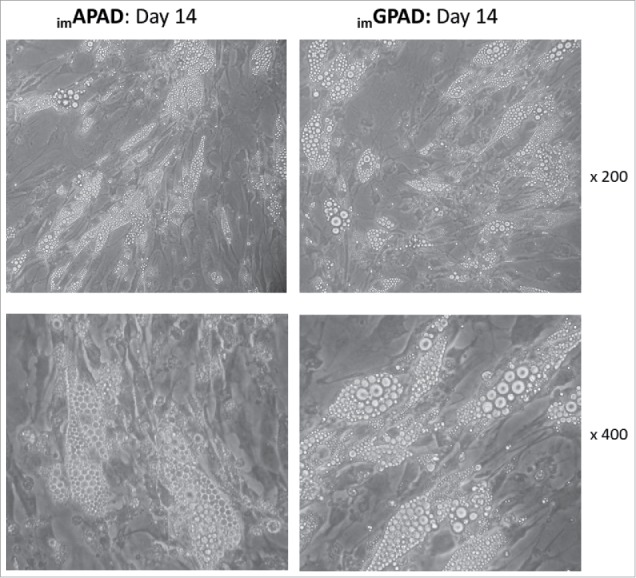
Light microscopy of _im_APAD and _im_GPAD cell lines after adipogenic differentiation. Lipid droplet accumulation was visible in both _im_APAD and _im_GPAD cell lines (passage 8–9) following a 14 day adipogenic differentiation time-course (upper panels: x 200 magnification; lower panels: x 400 magnification).

**Figure 5.**
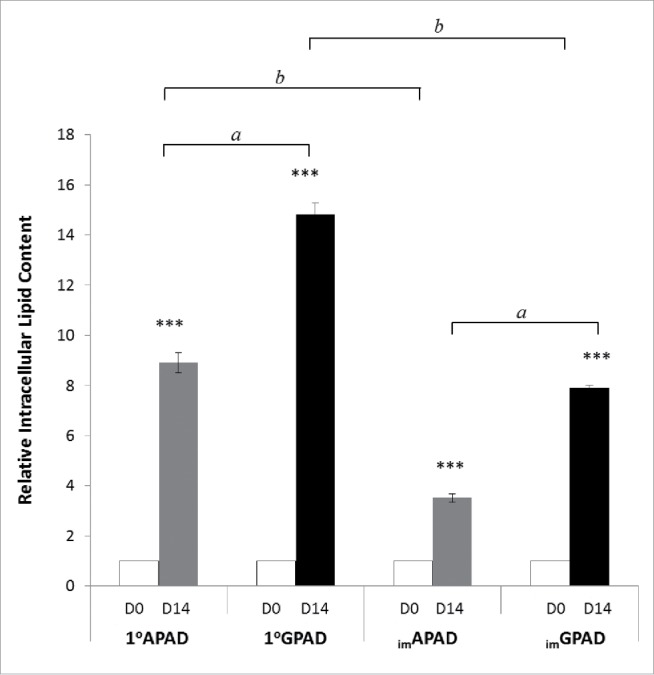
Intracellular lipid accumulation in _im_APAD and _im_GPAD cell lines. Lipid accumulation was measured by AdipoRed assay in _im_APAD and _im_GPAD cell lines (passage 10; n = 8) and 1°APAD and 1°GPAD cells (passage 8; n = 8). Lipid accumulation was determined on day 0 (D0) and day 14 (D14) of adipogenic differentiation and corrected for cell number. Data presented are relative to day 0 ± SEM. ***P < 0.0005; *^a^*APAD vs. GPAD cells, *^b^*primary vs. cell lines).

**Figure 6.**
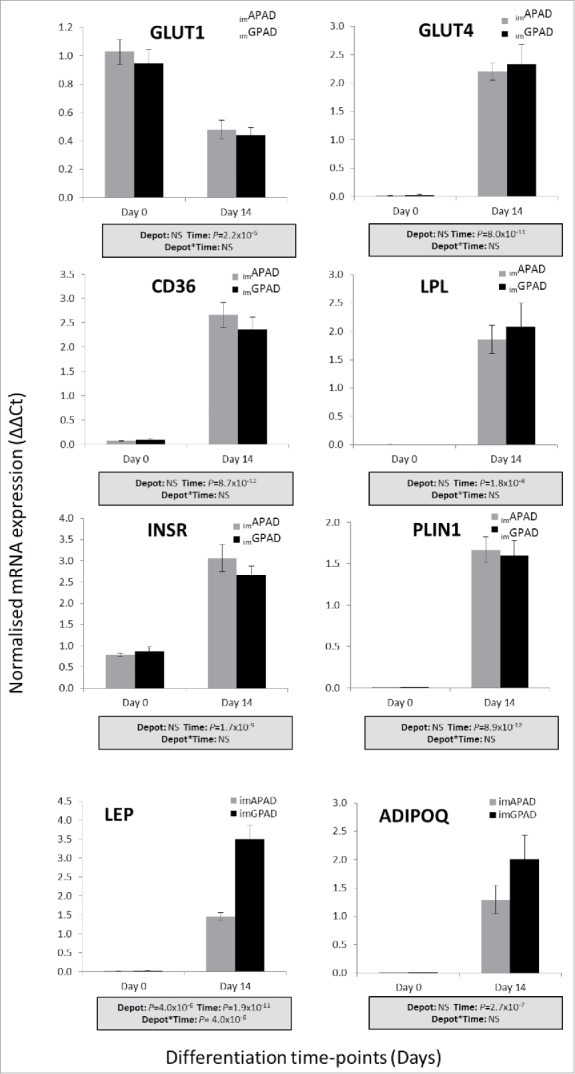
mRNA expression of genes involved in lipid and glucose metabolism in _im_APAD and _im_GPAD cell lines. mRNA expression of *INSR, GLUT1, GLUT4, CD36, LPL, PLIN1, ADIPOQ* and *LEP* at day 14 day of adipogenic differentiation was determined by real-time qPCR in _im_APAD and _im_GPAD cell lines (passage 8–9). Data are shown as ΔΔCt (normalized to *PPIA* and *PGK1*; n = 6, mean ± SEM). A multivariate general linear model was used to test for statistical significance between depots and time, and to assess depot × time interactions. *P*-values are presented in the shaded boxes, NS: non-significant.

Following adipogenic differentiation for 14 d intracellular lipid droplets were visible by light microscopy in both _im_APAD and _im_GPAD cell lines (passage 8–9) (Fig. 4). The final differentiated cell population was heterogeneous and contained some non-differentiated fibroblast-like cells in addition to the lipid-filled cells. This was also observed in the primary cells from which they were derived (Supplementary Fig. 4). Some subtle morphological differences were observed compared with the 1°APAD and 1°GPAD cells (passage 6–8). Specifically, the _im_APAD and _im_GPAD cell lines exhibited a more spindle-shaped phenotype and contained fewer lipid droplets compared with their differentiated primary counterparts (Fig. 4). Adipogenic capacity was assessed using a fluorescent lipophilic dye (AdipoRed) which labels intracellular lipids. Intracellular lipid accumulation markedly increased in both _im_APAD and _im_GPAD cells (3.5-fold and 7.9-fold, respectively) between day 0 (undifferentiated) and day 14 of differentiation (Fig. 5). In addition, markers of terminal adipocyte differentiation (*PLIN1, INSR, ADIPOQ, LEP, CD36* and *LPL*) were highly expressed at day 14 with no difference in expression observed between the 2 cell lines, except for *LEP* which showed significantly higher expression in _im_GPAD cells (Fig. 6). Notably, the _im_GPAD cells accumulated more lipid than the _im_APAD cells; this recapitulated the depot-difference in intracellular lipid accumulation observed between 1°APAD and 1°GPAD cells. Despite the robust adipogenic capacity displayed by the _im_APAD and _im_GPAD cell lines, total intracellular lipid accumulation was significantly lower (2.5-fold and 1.3-fold, respectively) when compared with the 1°APAD and 1°GPAD cells. Both _im_APAD and _im_GPAD cell lines continued accumulating lipids even at higher passages (passage 25), with _im_GPAD having more visible lipid droplets than _im_APAD (Supplementary Fig. 6).

Intracellular triglyceride (TAG) fatty acid composition of differentiated _im_APAD and _im_GPAD cells (passage 17) was examined by gas chromatography and revealed the principle fatty acid constituents in both cell lines to be: 16:0, 16–1*n-7*, 18:0, 18:1*n-9* and 18:1*n-7* (Table 1). Since no exogenous fatty acids were added to the culture medium the TAG composition was in keeping with synthesis and modification of these endogenous fatty acids by intrinsic DNL, Δ^9^desaturation and elongation pathways. Consistent with this, mRNA expression of key enzymes involved in each of these pathways (*ACACA, FASN, SCD, ELOVL3* and *ELOV5*) was readily detected in both _im_APAD and _im_GPAD preadipocytes (Table 1). Notably, _im_GPAD cells contained a higher proportion of 18:*1n-7* (vaccenic acid), the elongation product of Δ^9^ desaturase (SCD)-derived 16:1*n-7* (palmitoleic acid), compared with _im_APAD cells (Table 1). Fatty acid ratios were calculated as indices of SCD and elongase activity. Both 18:1*n-7*/16:0 and 18:1*n-7*/16:1*n-7* ratios were higher in _im_GPAD cells. *ELOVL3* gene expression was also significantly higher in _im_GPAD compared with _im_APAD at day 14, however there was no significant difference in *SCD* mRNA expression between _im_APAD and _im_GPAD cells. Finally, the _im_GPAD cells showed a tendency for higher total TAG content than the _im_APAD cells (Table 1) which was consistent with AdipoRed data from 1°APAD and 1°GPAD cells and earlier passage _im_APAD and _im_GPAD cells.

**Table 1.** TAG fatty acid composition and mRNA expression of lipid metabolism genes in _im_APAD and _im_GPAD cell lines at day 14.

	Glucose media		Glucose + fatty acid media	
	_im_APAD	_im_GPAD	_im_APAD	_im_GPAD
*Fatty acid (mol %)*				
14:0	4.80 ± 1.00	2.70 ± 0.10	0.50 ± 0.07	1.00 ± 0.04*
16:0	36.2 ± 3.90	28.3 ± 0.30	27.2 ± 0.13	26.0 ± 0.15*
16:1*n*-7	9.30 ± 0.40	10.3 ± 0.90	2.00 ± 0.04	4.20 ± 0.10*
18:0	10.1 ± 0.60	7.00 ± 0.8	2.20 ± 0.01	2.60 ± 0.02*
18:1*n*-9	28.0 ± 2.60	27.5 ± 0.80	50.6 ± 0.10	51.8 ± 0.24
18:1*n*-7	9.90 ± 0.12	22.7 ± 0.12*	N.D.	N.D
18:2*n*-6	3.30 ± 0.80	1.60 ± 0.40	17.6 ± 0.08	14.4 ± 0.19*
*Total fatty acids (µmol/l)*	37.0 ± 1.4	100.8 ± 29.5	352.7 ± 28.0	456.7 ± 5.6*
*Fatty acid ratios*				
16:1*n*-7/16:0	0.26 ± 0.03	0.36 ± 0.04	0.07 ± 0.002	0.16 ± 0.005*
18:1 *n*-7/16:0	0.29 ± 0.06	0.80 ± 0.05*	N.D.	N.D.
18:1 *n*-7/16:1*n*-7	1.06 ± 0.09	2.22 ± 0.10*	N.D.	N.D.
18:0/16:0	0.28 ± 0.02	0.25 ± 0.03	0.08 ± 0.001	0.10 ± 0.001*
[*^13^C*] *DNL calculations*				
16:0 content^1^	1.73 ± 0.25	3.64 ± 1.04	12.3 ± 1.03	15.2 ± 0.27
[^13^C]16:0 content^1^	0.21 ± 0.01	0.55 ± 0.05*	0.30 ± 0.05	0.62 ± 0.06*
[^13^C]16:0 carbon label (%)	12.5 ± 1.07	16.5 ± 2.69	2.50 ± 0.56	4.06 ± 0.37*
*mRNA expression*				
* ACACA*	2.35 ± 0.11	2.36 ± 0.17	1.51 ± 0.08	1.63 ± 0.07*
* FASN*	2.25 ± 0.19	2.30 ± 0.33	1.29 ± 0.18	1.06 ± 0.16
*SCD*	1.88 ± 0.23	2.13 ± 0.38	1.25 ± 0.20	1.20 ± 0.06
*ELOVL3*	0.22 ± 0.03	0.45 ± 0.03*	0.20 ± 0.01	0.36 ± 0.03*
*ELOVL5*	0.63 ± 0.04	0.48 ± 0.08	0.56 ± 0.05	0.34 ± 0.01*

*Note*. Cells were differentiated in media containing 17.5 mM glucose or 17.5 mM glucose + 200µM fatty acid mixture (45% oleate, 30% palmitate, 25% linoleate). 12.5 mM of the final glucose concentration was isotopically labeled with [U-^13^C] to assess *de novo* lipogenesis (DNL); ^1^ µg per 4 × 10^5^ cells. Data are presented as means ± *SEM* (n = 3). *P < 0.05; _im_APAD vs. _im_GPAD; paired samples *t-*test.

To examine handling of exogenous fatty acids by the _im_APAD and _im_GPAD cells a fatty acid mixture comprising of oleate:palmitate:linoleate (45:30:25%) was added to the culture medium for the last 7 d of differentiation. This led to a significant increase in intracellular TAG content in the _im_APAD (5.0 ± 0.2 vs. 48.3 ± 3.8 µg/ 4 × 10^5^ cells; *P* < 0.05) and _im_GPAD cell lines (13.6 ± 4.0 vs. 62.4 ± 0.7 µg/ 4 × 10^5^ cells; *P* < 0.05). Despite both being exposed to the same fatty acid mixture there were significant differences in TAG fatty acid composition between _im_APAD and _im_GPAD cells; 95% of _im_APAD TAG was accounted for by the exogenous fatty acids. By comparison TAG in _im_GPAD cells comprised 92% exogenous and 8% endogenous fatty acids. Specifically, 14:0 and 16:1*n-7* were 2-fold higher in _im_GPAD compared with _im_APAD cells. Consistent with a higher capacity for synthesis of endogenous fatty acids the rate limiting enzyme for DNL, *ACACA*, was found to be more highly expressed in _im_GPAD cells than _im_APAD cells, as was *ELOVL3* (Table 1).

To further examine differences in DNL between the _im_APAD and _im_GPAD cells isotopically labeled D-[U-^13^C] glucose was added to the differentiation medium, and ^13^C-enrichment of TAG-16:0 was measured at day 14 by gas-chromatography mass-spectrometry (GC-MS). In line with previous work,^38^ the mass isotopomer spectrum showed greater labeling from M+1 to M+5, with decreased higher labeling up to M+16; the proportion of TAG-16:0 derived from [U-^13^C] glucose was calculated as 12.5% in _im_APAD cells and 16.5% in _im_GPAD cells (Table 1). This equated to a significantly (2-fold) higher total amount of ^13^C-labeled TAG-16:0 in _im_GPAD cells compared with _im_APAD cells (Table 1). No differences in expression of glucose transporters*, GLUT1* and *GLUT4*, were observed between _im_APAD and _im_GPAD cells (Fig. 6). In experiments where exogenous fatty acids were added to the differentiation medium ^13^C-labeled TAG-16:0 was also 2-fold higher in _im_GPAD cells. Overall these data suggest that the _im_GPAD cells possess a higher intrinsic capacity for DNL, Δ^9^ desaturation and 16:1*n-7* elongation than the _im_APAD cells.

### Catecholamine-stimulated lipolysis in _im_APAD and _im_GPAD cells

**Figure 7.**
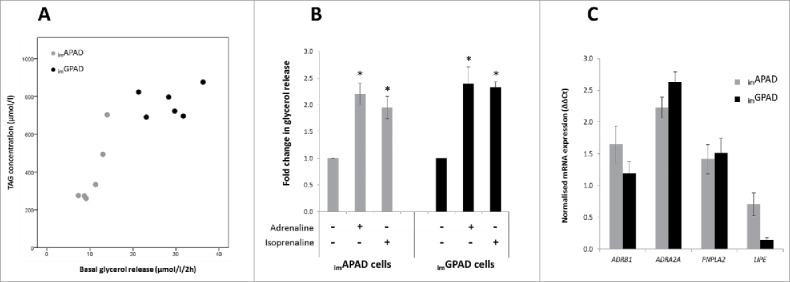
Lipolysis in _im_APAD and _im_GPAD cell lines. (A) Basal glycerol release plotted against total TAG content in _im_APAD and _im_GPAD cell lines (passage 16–17). (B) Catecholamine-stimulated glycerol release in _im_APAD and _im_GPAD cells relative to basal glycerol release. Cells were treated with 5 mM KRH buffer containing either adrenaline (100nM) or isoprenaline (100nM) (n = 3, mean ± SEM, normalized for total TAG content; *P < 0.05, Wilcoxon signed-rank). (C) mRNA expression of adrenergic receptors and lipases at day 14 of adipogenic differentiation in _im_APAD and _im_GPAD cells (passage 8–9). Data are shown as ΔΔCt values (normalized to *PPIA* and *PGK1*; n = 6, mean ± SEM).

To further examine functionality of the *in vitro* differentiated _im_APAD and _im_GPAD cell lines (passage 16–17) glycerol release was determined as a direct measure of lipolysis (Fig. 7). Basal lipolysis was approximately 2-fold higher in the _im_GPAD cells compared with _im_APAD cells and this tracked closely with intracellular TAG content (Fig. 7A). Therefore catecholamine-stimulated glycerol release data were normalized to intracellular TAG content. The addition of adrenaline (100nM) or isoprenaline (100nM) induced a significant 2-fold increase in the release of glycerol into the culture medium compared with baseline levels (Fig. 7B). No significant difference in glycerol release was observed between the _im_APAD and _im_GPAD cell lines. Consistent with this finding, mRNA expression of adrenergic receptors (*ADRB1* and *ADRA2A*) and adipose tissue lipases (*PNPLA2* and *LIPE*) was not significantly different between the _im_APAD and _im_GPAD cells (Fig. 7C).

### Inherent depot-specific developmental gene expression in _im_APAD and _im_GPAD cell lines

**Figure 8.**
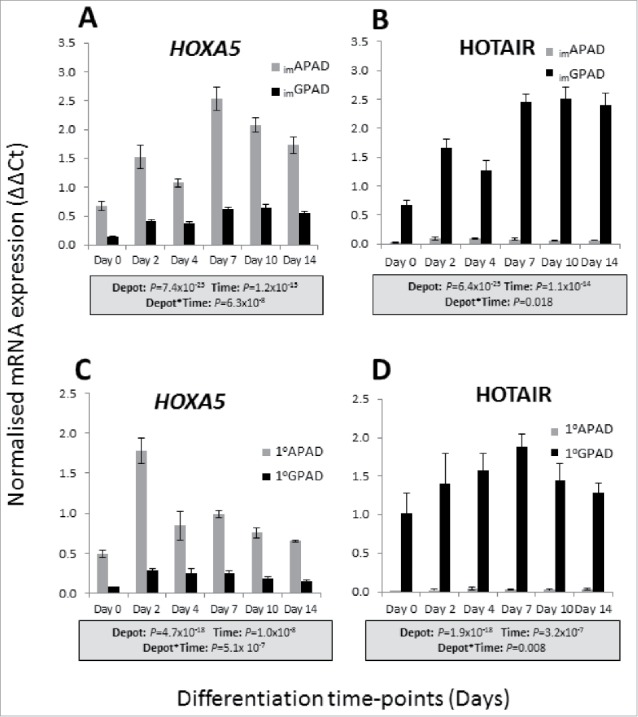
Inherent depot-specific expression of developmental genes in _im_APAD and _im_GPAD cells. mRNA expression of *HOXA5* and *HOTAIR* over a 14 day adipogenic differentiation time-course was determined by real-time qPCR in _im_APAD and _im_GPAD cell lines (A and B) and 1°APAD and 1°GPAD cells (C and D). Data are shown as ΔΔCt (normalized to *PPIA* and *PGK1*; n = 6, mean ± SEM). A multivariate general linear model was used to test for statistical significance between depots and time, and to assess depot x time interactions. *P*-values are presented in the shaded boxes, NS: non-significant.

We previously reported that undifferentiated _im_APAD and _im_GPAD cell lines retain inherent depot-specific mRNA expression patterns which mirror those seen in whole ASAT and GSAT.^39^ Here we selected 2 of the previously reported genes with the most differential expression (*HOXA5* and *HOTAIR*) and examined their expression pattern across a differentiation time-course in _im_APAD and _im_GPAD cell lines (passage 8–9) compared with 1°APAD and 1°GPAD cells (passage 6–8). Both *HOXA5* and *HOTAIR* displayed strong depot-specific expression between the _im_APAD and _im_GPAD cell lines (Fig. 8A and B); *HOXA5* expression was significantly higher in _im_APAD compared with _im_GPAD whereas the opposite was observed for *HOTAIR*. Both genes were significantly upregulated over the differentiation time course in _im_APAD and _im_GPAD, suggesting a regulated role in adipogenesis. The depot-specific expression patterns were retained across the entire 14 day differentiation time-course, and were consistent with expression patterns observed in the 1°APAD and 1°GPAD cells (Fig. 8C and D). Depot-specific expression of *HOXA5* and *HOTAIR* was retained in _im_APAD and _im_GPAD cells up to passage 30 indicating that depot differences between _im_APAD and _im_GPAD cells are maintained across multiple cell generations (Supplementary Fig. 5A and B).

## Discussion

The _im_APAD and _im_GPAD cell lines reported herein represent a novel tool to aid the investigation of depot-specific adipocyte function. The disparity in metabolic disease risk conferred by GSAT and ASAT accumulation in humans remains largely unexplained.^3,6^ Consequently, there is an obvious need to explore molecular mechanisms controlling adipocyte commitment, differentiation and function in these contrasting WAT depots. Nonetheless, research in this field has been hampered by the lack of a suitable human *in vitro* adipogenic model and, to our knowledge, there is currently no paired ASAT-GSAT human preadipocyte cell line in use.

The _im_APAD and _im_GPAD cell lines, which co-express hTERT and HPV16-E7, display enhanced proliferation rates and continue to divide even at passage 30 whereas primary cells fail to proliferate after passage 14. The _im_APAD and _im_GPAD cell lines also retain the capacity for white adipogenic differentiation and *de novo* synthesis of fatty acids. Thus they overcome some of the main limitations of primary human preadipocyte cultures i.e., limited supply of human WAT, a finite capacity for expansion and loss of adipogenic capacity when cultured *in vitro*. It should be noted that the capacity for adipogenic differentiation is modestly reduced when compared with that of 1° APAD and 1° GPAD cells. The reason for this is not entirely clear, however, HPV-E7 has been described to inhibit activity of the glycolytic enzyme pyruvate kinase.^40^ This rate-limiting enzyme regulates the amount of glucose available for *de novo* lipogenesis and thus may explain the lower lipid accumulation in the immortalised cells compared with the primary cells.

Most importantly the _im_APAD and _im_GPAD cells retain intrinsic functional characteristics of their depot-of-origin. Specifically, _im_GPAD cells displayed a higher incorporation of ^13^C-labeled glucose into TAG-palmitate suggesting they possess a higher capacity for DNL than the _im_APAD cells. This is consistent with higher mRNA expression of *ACACA* in the _im_GPAD cells and mirrors the depot difference in lipid accumulation that was seen in the originating primary cells. Whether differences in insulin-stimulated glucose uptake also contribute to the higher incorporation of ^13^C-glucose in _im_GPAD cells cannot be excluded. However, there were no significant depot differences in expression of *GLUT1, GLUT4* or *INSR*. Furthermore, TAG synthesized by _im_GPAD cells was more enriched for monounsaturated fatty acids (16:1*n-7* and 18:1*n-7*) derived from SCD (Δ^9^-desaturase) and elongase activity than TAG in the _im_APAD cells. Similar findings were previously reported in human primary preadipocytes isolated from different donors.^21^ The DNL pathway is tightly coupled with these fatty acid modification pathways in adipocytes,^41^ and our findings of higher fatty acid flux through SCD and elongation pathways in _im_GPAD cells is consistent with the reported fatty acid composition of *in vitro* differentiated primary preadipocytes and adipose tissue samples from GSAT and ASAT.^21,42^ A potential role for 16:1*n-7* as an insulin-sensitizing lipokine has previously been proposed although evidence supporting this role in humans remains weak.^43,44,45^ Nevertheless, the finding that GSAT-derived preadipocytes retain intrinsic mechanisms favoring synthesis of SCD-derived fatty acids highlights notable differences in endogenous fatty acid metabolism between GSAT and ASAT. This functional attribute may in turn be of importance for understanding the differential metabolic risk conferred by these 2 WAT depots, which requires further mechanistic investigation.

The _im_APAD and _im_GPAD cell lines retain striking depot-specific expression of 2 developmental transcription factors involved in embryogenesis and organ development (*HOXA5, HOTAIR)*. The *HOXA5* and *HOTAIR* expression patterns mirror those seen in the originating primary preadipocytes and are consistent with depot-specific expression patterns observed in whole ASAT and GSAT.^26,27^ These genes have also been implicated in regulation of adipose tissue function.^46,47^ Therefore differential expression of *HOXA5* and *HOTAIR* may contribute to functional differences seen between depots. These observations support the view that preadipocyte populations from different WAT depots are inherently distinct with depot-specific gene expression likely under epigenetic control. Indeed, we have previously reported depot-specific DNA methylation patterns of several developmental genes (*HOTAIR, TBX5*) which regulate adipogenesis in the _im_APAD and _im_GPAD cells.^27^ Thus these cell lines may also prove useful for investigating epigenetic regulation of regional adipocyte biology. Furthermore, inherent differences in components of the WNT-signaling pathway have recently been described between the _im_APAD and _im_GPAD cell lines.^48^ Notably, in these experiments, knockdown of the Wnt co-receptor, LRP5, led to opposite effects on adipocyte differentiation in _im_APAD and _im_GPAD cells. These experiments demonstrate the usefulness of the _im_APAD and _im_GPAD cells for genetic modification studies. It also highlights the importance of performing studies in paired cells rather than cells derived from a single WAT depot when investigating the molecular mechanisms of body fat distribution.

As well as expressing the basic transcriptional machinery required for successful adipogenic differentiation, the *in vitro* differentiated _im_APAD and _im_GPAD cells responded to catecholamine stimulation with a 2-fold induction in glycerol release. This response was similar in magnitude to that reported for the Chub-S7 human preadipocyte cell line which derives from ASAT and also co-expresses hTERT and HPV16-E7.^35^ It is well established that human gluteal or femoral adipocytes are less responsive to catecholamine-stimulated lipolysis than subcutaneous abdominal adipocytes.^19,49-52^ Specifically, gluteofemoral adipocytes are resistant to adrenaline induced-lipolysis while retaining a robust lipolytic response to the β-selective agonist isoprenaline. However, here we report no apparent depot difference in lipolytic response to adrenaline or isoprenaline between the _im_APAD and _im_GPAD cells. It should be noted that previous findings were exclusively based on freshly isolated mature adipocytes^50-52^ or on *in vivo* measurements of adipose tissue lipolysis.^19,49^ Whether depot differences in lipolysis are an intrinsic feature of *in vitro* differentiated preadipocytes has not been demonstrated. The resistance of gluteal adipocytes to adrenaline has been attributed to an increased anti-lipolytic (α-adrenergic) responsiveness.^19,50,51^ Although we observed a tendency for higher *ADRA2A* expression in _im_GPAD cells the depot differences in gene expression were considerably blunted in comparison to those reported for whole tissue biopsies^19^ and freshly isolated adipocytes.^53^ Furthermore, loss of α-adrenergic anti-lipolytic responsiveness has previously been observed for *in vitro* differentiated primary human preadipocytes, suggesting the loss of depot-specific differences in lipolysis may be a consequence of the *in vitro* culture conditions, ^54^ for example the absence of a local paracrine and/or endocrine factor(s). The dose of agonist selected for _im_APAD and _im_GPAD lipolysis studies (10^−7^M) had previously been shown to induce maximal lipolysis in human abdominal and gluteal adipocytes *in vitro* and depot-specific glycerol release was also reported at this dose.^30,50^ However, it is possible that this concentration is suboptimal for detecting depot differences between the _im_APAD and _im_GPAD cells and further studies are required to investigate this.

In summary, we have generated _im_APAD and _im_GPAD cell lines which are a novel *in vitro* model for investigating depot-specific preadipocyte function. Elucidating the mechanisms that underlie the different risk profiles and metabolic profiles of ASAT and GSAT is important for developing targeted therapies for obesity and its related metabolic disease.

## Methods

### Isolation and culture of human primary white and brown preadipocytes

Ethical approval for adipose biopsies was granted by Oxfordshire Clinical Research Ethics Committee (WAT study: 08/H0606/107 and brown adipose tissue study: 12/SC/0446) and the study participants gave written informed consent. WAT biopsies were obtained from a 50-year old healthy male (BMI: 24.4 kg/m^2^) from the Oxford Biobank (http://www.oxfordbiobank.org.uk) who had given consent to be re-approached for research purposes. Paired WAT biopsies were taken under local anesthetic (1% lignocaine) by needle aspiration at the level of the umbilicus (ASAT) and from the upper-, outer-quadrant of the gluteal region (GSAT). Primary preadipocytes (1°) were isolated from ASAT and GSAT biopsies as described previously.^41,55^ Preadipocytes were cultured in Dulbecco's modified Eagle's medium/F12 Ham's nutrient mixture (v/v, 1:1) containing 17.5 mM glucose and supplemented with 10% foetal calf serum, 0.25 ng/ml fibroblast growth factor, 2 mM glutamine, 100 units/ml penicillin and 100 µg/ml streptomycin. Cell stocks of 1° APAD and 1° GPAD cells were prepared and stored in liquid nitrogen for future studies. A brown adipose tissue biopsy was obtained from a 36-year old male patient undergoing thyroid carcinoma surgery (BMI >30). Preadipocytes were isolated and cultured using the same protocol described above.

### Generation of preadipocyte cell lines

The _im_APAD and _im_GPAD preadipocyte cell lines were generated according to the method of Darimont *et al.*^35^ with additional modifications. Briefly, human telomerase reverse transcriptase (hTERT) and human papillomavirus type 16 E7 oncoprotein (HPV16-E7) were sub-cloned into the pLenti6.3/V5-DEST lentiviral expression vector (Invitrogen) from the pBABE-neo-hTERT and pGEX2T E7 plasmids (Addgene), respectively. For the constitutive expression of hTERT and HPV16-E7 lentiviral particles were generated in 293FT producer cells using the ViraPower HiPerform Lentiviral Expression System (Invitrogen). 1° APAD and 1° GPAD cells were pre-treated with hexadimethrine bromide (8 µg/ml) and then transduced with hTERT lentiviral particles. To select preadipocytes constitutively expressing hTERT cells were cultured in the presence of blasticidin (2 µg/ml). Blasticidin treatment of non-transduced cells was used to determine the optimal lethal concentration of blasticidin. Blasticidin-resistant cells were then transduced with HPV16-E7 lentiviral particles. Expression of hTERT and HPV16-E7 was driven by the human cytomegalovirus (CMV) immediate early promoter within the pLenti6.3/V5 vector. _im_APAD and _im_GPAD cells were cultured as described for human primary preadipocytes with the addition of blasticidin (2 µg/ml) to the culture medium.

### Western blot analysis

Protein expression of hTERT and HPV16-E7 was confirmed by Western blotting. Cell lysates were prepared in ice-cold lysis buffer containing 50 mM Tris pH8.0, 250 mM NaCl, 5 mM EDTA, 0.5% Igepal CA-630, 10 mM sodium fluoride, 1 mM sodium orthovanadate and protease inhibitors (Complete EDTA-free, Roche). Equal amounts of protein (25 µg) were resolved by SDS-PAGE, transferred onto polyvinylidene fluoride membrane (Bio-Rad) and immunoblotted with the following antibodies: anti-HPV16-E7 (1:200, sc-51951: Santa Cruz Biotechnology), anti-hTERT (1:500, Y050419: Applied Biological Materials Inc.), anti-actin (1:2000, sc-1616: Santa Cruz Biotechnology) followed by horseradish peroxidise-conjugated secondary antibodies: goat anti-rabbit IgG (1:2000, #170–6515: Bio-Rad) and goat anti-mouse IgG (1:1000, P0447: Dako). Detection was performed by enhanced chemiluminescence (GE Healthcare).

### Telomerase activity

Telomerase activity was measured using the TRAPeze XL Telomerase Detection kit (Millipore; S7707). 1 × 10^6^ cells were lysed in CHAPS lysis buffer. Sample preparation and PCR amplification was performed according to the manufacturer's instructions. Each sample extract was assayed in duplicate either with or without heat treatment (85°C) to provide a negative control. PCR yield was determined by measuring fluorescent signal (excitation: 485 nm and emission: 530 nm) on a CytoFluor Multi-Well Plate Reader series 4000 (PerSeptive Biosystems).

### Proliferation

The _im_APAD / _im_GPAD cell lines (passage 9 – 30) and 1° APAD / 1° GPAD cells (passage 9 – 14) were seeded at a low density (2000 cells/cm^2^)and maintained in growth medium for 4 d at which point the cells were still sub-confluent. Cells were trypsinised and counted manually using a haemocytometer or using an automated Cellometer Auto T4 (Nexcelom Bioscience). Doubling time (DT) was calculated as previously described^48^ using the following formula: DT = T × ln2/ln(cells^end^/cells^start^) where T = culture time (days).

### Adipogenic differentiation culture conditions

Fully confluent white preadipocytes were stimulated for 14 d with an adipogenic cocktail consisting of Dulbecco's modified Eagle's medium/F12 Ham's nutrient mixture (v/v, 1:1), 17.5 mM glucose, 2 mM glutamine, 17 μM pantothenate, 100 nM human insulin, 10 nM triiodo-L-thyronine, 33 μM biotin, 10 μg/ml transferrin, 1 μM dexamethasone, 100 units/ml penicillin and 100 µg/ml streptomycin. Troglitazone (4 μM) and 3-isobutyl-1-methylxanthine (0.25 mM) were added to the adipogenic medium for the first 4 d. For experiments using exogenous fatty acids, a fatty acid mixture (200 µM) comprising oleate:palmitate:linoleate (45:30:25%) bound to BSA was added to the differentiation medium from day 7 onwards and troglitazone (4 μM) was included throughout the whole time course. Fully confluent brown preadipocytes were differentiated according to the protocol above for 14 d. Cells were then stimulated with forskolin (10 µM) for 4 h and collected for further analyses.

### Gene expression analysis

cDNA was synthesized from 500 µg of total RNA extracted from preadipocytes using the High Capacity cDNA Reverse Transcription Kit (Life Technologies, UK). qPCR was performed on a 1/40 cDNA dilution using Taqman Assays-on-Demand (Applied Biosystems) and Kapa Probe Fast Mastermix (Kapa Biosystems) on an ABI Prism 7900HT. Reactions were performed in a final volume of 6µl in triplicate. Relative transcript expression was calculated using the ΔΔCt relative quantification method^56^ where *ΔCt = Assay efficiency^(^^min CT- Sample^^CT^^)^*. The ΔCT values of target genes were normalized to the ΔCt (geometric mean) of reference transcripts *PPIA* and *PGK1*.^57^

### Measurement of intracellular lipid content

Intracellular lipid was measured in preadipocytes using the AdipoRed Assay (Lonza) according to the manufacturer's instructions. Fluorescence was measured (excitation: 485 nm and emission: 580 nm) on a CytoFluor Multi-Well Plate Reader series 4000 (PerSeptive Biosystems).

### Gas chromatography (GC) analysis of TAG fatty acids

Total lipids were extracted from differentiated preadipocytes at day 14,^58^ TAG separated and fatty acid methyl esters (FAMEs) prepared and analyzed by GC as previously described.^41^ Analytic accuracy was assessed using external and in-house quality control samples (AOCS std#6, Thames Restek UK Ltd; Seven Seas Ltd, Hull, UK). Known amounts of an internal standard (glyceryl pentadecanoate (15:0) were added to samples before extraction. Fatty acid concentrations were calculated relative to the internal standard and results were expressed either as µg of fatty acid per 4 × 10^5^ cells or as a mole percentage. Fatty acid product to precursor ratios were calculated from mole percentages as indices of SCD activity (16:1*n-7*/16:0), elongase activity (18:1*n-7*/16:1*n-7* and 18:0/16:0) and combined SCD and elongase activity (18:1*n-7*/16:0).

### Measurement of [^13^C]-glucose incorporation into TAG-palmitate

Differentiation medium containing 5 mM glucose and 12.5 mM isotopically [U-^13^C]-labeled glucose (CK Gas, Cambridgeshire, UK) was added to cells throughout differentiation to determine the contribution glucose makes for DNL. After lipid extraction and separation of the TAG fraction the incorporation of ^13^C (from glucose) into TAG palmitate was measuring using GC-MS with monitoring ions with mass-to-charge ratios (*m/z*) of 270.3 (M+0) through to 286.3 (M+16).^38,59^ Relative abundance values were corrected for natural abundance in cells that were ^13^C-glucose naïve. The percentage of ^13^C-labeled carbon atoms in palmitate was determined by quantitative mass spectral analysis (QMSA) calculations were based on the method described by Tayek and Katz, 1996.^60^ Briefly, we calculated the fraction of all carbon atoms in palmitate that were ^13^C labeled (from the precursor ^13^C-glucose) as described.^38^

### Lipolysis

Differentiated (day 14) preadipocytes were cultured for 24 h before experimentation in DMEM/F12 Ham containing 5 mM glucose, 10% foetal calf serum, 100 units/ml penicillin and 0.1 mg/ml streptomycin. On the experimental day cells underwent a 2 h washout period in Krebs Ringer HEPES (KRH) buffer containing 5 mM glucose and 3.5% bovine serum albumin. The cells were then incubated for 2 h in fresh KRH buffer (basal) followed by a 2 h incubation in KRH buffer containing either adrenaline (100 nM) or isoprenaline (100 nM). Samples were collected at the end of each 2 h incubation period and glycerol measurements were made using an enzymatic assay (GY105, Randox Laboratories Ltd). At the end of the experiment cells were harvested in lysis buffer containing 1% IGEPAL CA-630, 150 mM NaCl and 50 mM Tris-HCl (pH8.0). Cell lysates were sonicated and heated at 95°C for 30 minutes. Cooled lysates were centrifuged at 12,000 g for 10 minutes and cellular TAG concentration was measured by enzymatic assay (TAG assay, Instrumentation Laboratory UK). Glycerol and TAG assays were both run on an ILAB 650 clinical analyzer (Instrumentation Laboratory UK). Glycerol measurements were normalized to cellular TAG content.

### Statistical methods

Statistical analyses were performed in SPSS 20.0. Statistical tests used were as described in the results section.

## Supplementary Material

KADI_A_1277052_Supplemental.docx

## Abbreviations

1° APAD: primary abdominal preadipocytes
1° GPAD: primary gluteal preadipocytes
ASAT: abdominal subcutaneous adipose tissue
DNL: *de novo* lipogenesis
GSAT: gluteal subcutaneous adipose tissue
_im_APAD: immortalised abdominal preadipocytes
_im_GPAD: immortalised gluteal preadipocytes
TAG: triacylglycerol
WAT: white adipose tissue
