# Supplementary material for: A cellular model for the investigation of depot specific human adipocyte biology
Source: Adipocyte. 2017 Jan 6;6(1):40–55. doi: 10.1080/21623945.2016.1277052 (PMC5358705; doi:10.1080/21623945.2016.1277052)
Supplement: KADI_A_1277052_Supplemental.docx [file kadi-06-01-1277052-s001.docx]

**SUPPLEMENTARY FILES**

**Supplementary Figure Legends**

**Supplementary Figure 1: Loss of proliferative capacity in 1^o^APAD and 1^o^GPAD cells.** Proliferation rates of paired _im_APAD and _im_GPAD cell lines (passage 15) were compared to paired 1^o^APAD and 1^o^GPAD preadipocytes (passage 14). 1^o^APAD and 1^o^GPAD failed to proliferate whereas the _im_APAD and _im_GPAD cell lines continued to exhibit enhanced rates of proliferation.

**Supplementary Figure 2: mRNA expression of adipogenic markers in 1^o^APAD and 1^o^GPAD** **cells**. mRNA expression of *PPARG2, CEBPA, CEBPB, CEBPD, PLIN1* and *ADIPOQ* over a 14 day adipogenic differentiation time-course was determined by **real-time qPCR in** 1^o^APAD and 1^o^GPAD **cells (passage 6-8).** Data are shown as ΔΔCt (normalised to *PPIA* and *PGK1*; n=6, mean ± SEM). A multivariate general linear model was used to test for statistical significance between depots and time, and to assess depot x time interactions. *P*-values are presented in the shaded boxes, NS: non-significant.

**Supplementary Figure 3: mRNA expression of adipogenic markers in high passage _im_APAD** **and** **_im_GPAD cell lines**. mRNA expression of *PPARG2* and *CEBPA* over a 14 day adipogenic differentiation time-course was determined by **real-time qPCR in** _im_APAD and _im_GPAD **cells (passage 17-21).** Data are shown as ΔΔCt (normalised to *PPIA* and *PGK1*; n=6, mean ± SEM). A multivariate general linear model was used to test for statistical significance between depots and time, and to assess depot x time interactions. *P*-values are presented in the shaded boxes, NS: non-significant.

**Supplementary Figure 4: Light microscopy of** **1^o^APAD and 1^o^GPAD** **cells after adipogenic differentiation.** Lipid droplet accumulation was visible in both 1^o^APAD and 1^o^GPAD cells (passage 6-8) following a 14 day adipogenic differentiation time-course (upper panels: x 200 magnification; lower panels: x 400 magnification).

**Supplementary Figure 5: Inherent depot-specific expression of developmental genes in _im_APAD and _im_GPAD cells.**  mRNA expression of *HOXA5* **(A)** and *HOTAIR* **(B)** over a 14 day adipogenic differentiation time-course was determined by **real-time qPCR in** _im_APAD and _im_GPAD **cell lines** (passage 30)**.** Data are shown as ΔΔCt (normalised to *PPIA* and *PGK1*; n=6, mean ± SEM). A multivariate general linear model was used to test for statistical significance between depots and time, and to assess depot x time interactions. *P*-values are presented in the shaded boxes, NS: non-significant.

**Supplementary Figure 6: Light microscopy of _im_APAD and _im_GPAD cells after adipogenic differentiation.** Lipid droplet accumulation was visible in both **in** _im_APAD and _im_GPAD **cells** (passage 25) following a 14 day adipogenic differentiation time-course (upper panels: x 200 magnification; lower panels: x 400 magnification).

**Supplementary Figure 1:**

**
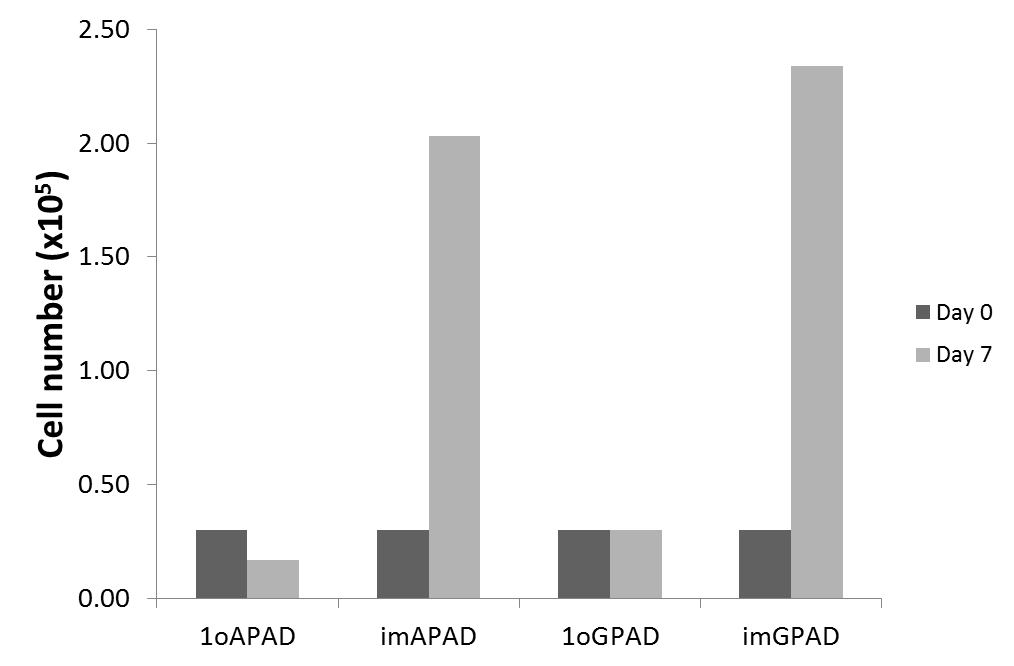
**

**
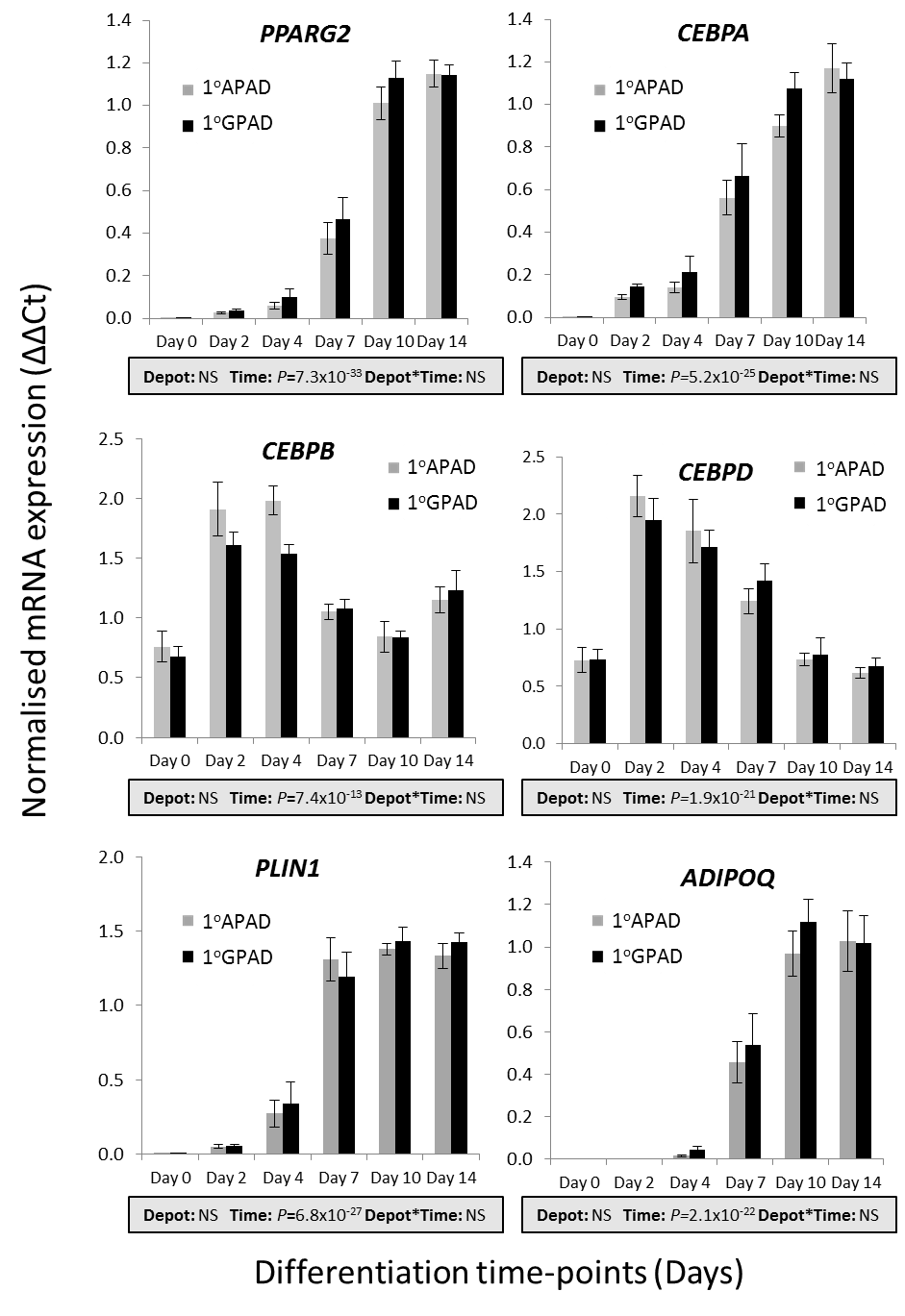
Supplementary Figure 2:**

**Supplementary Figure 3:**

**
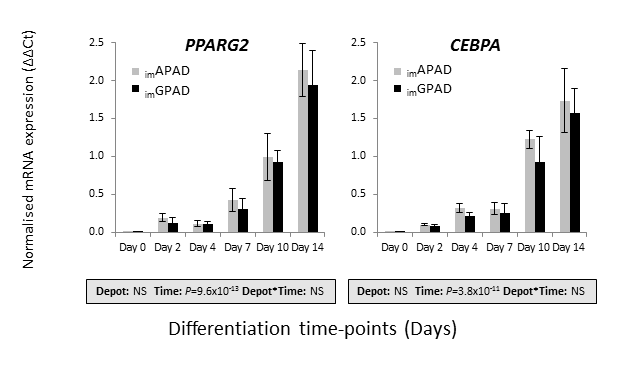
**

**Supplementary Figure 4:**

**
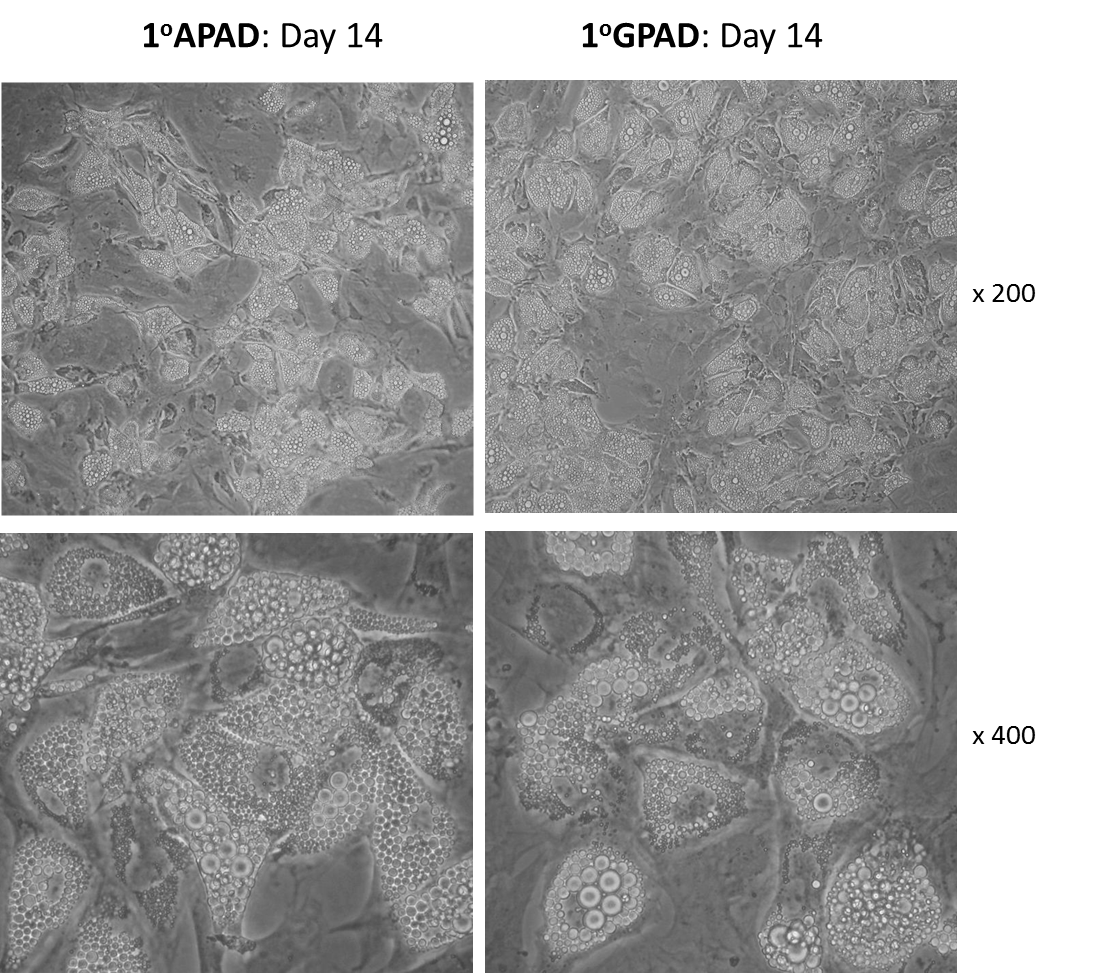
**

**Supplementary Figure 5:**


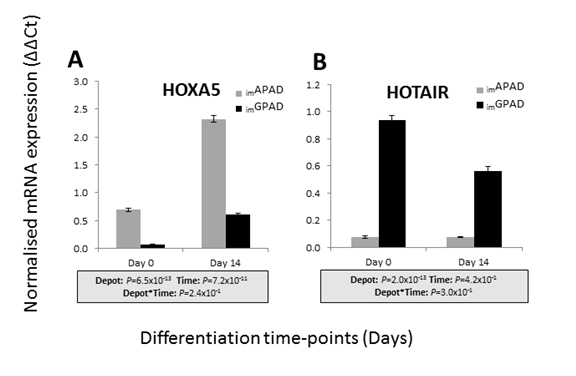


**Supplementary Figure 6:
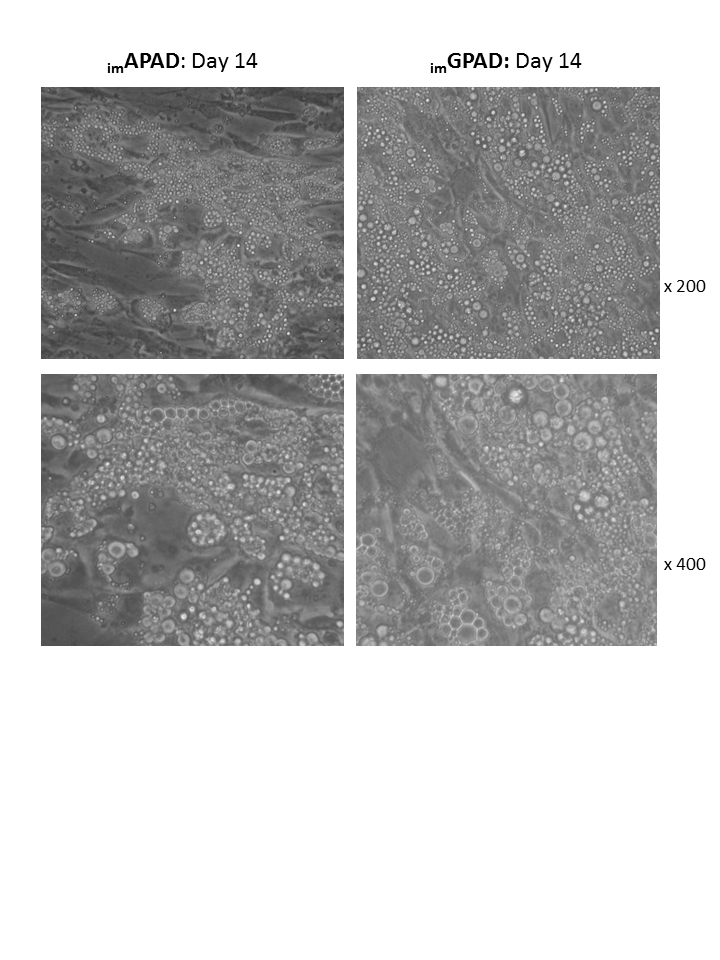
**
